# Real-world data and the patient perspective: the PROmise of social media?

**DOI:** 10.1186/s12916-018-1247-8

**Published:** 2019-01-16

**Authors:** Laura McDonald, Bill Malcolm, Sreeram Ramagopalan, Hayley Syrad

**Affiliations:** 1 Centre for Observational Research and Data Sciences, Bristol-Myers Squibb, Uxbridge, UK; 2 Bristol-Myers Squibb, Uxbridge, UK; 3 Evidera, London, UK

**Keywords:** Social media, Patient-reported outcomes, Epidemiology, Real-world data, Patient-centricity

## Abstract

Understanding the patient perspective is fundamental to delivering patient-centred care. In most healthcare systems, however, patient-reported outcomes are not regularly collected or recorded as part of routine clinical care, despite evidence that doing so can have tangible clinical benefit. In the absence of the routine collection of these data, research is beginning to turn to social media as a novel means to capture the patient voice. Publicly available social media data can now be analysed with relative ease, bypassing many logistical hurdles associated with traditional approaches and allowing for accelerated and cost-effective data collection. Existing work has shown these data can offer credible insight into the patient experience, although more work is needed to understand limitations with respect to patient representativeness and nuances of captured experience. Nevertheless, linking social media to electronic medical records offers a significant opportunity for patient views to be systematically collected for health services research and ultimately to improve patient care.

## Patient-centricity in real-world research: prioritising the patient perspective

Real-world data (RWD) are those data collected outside conventional randomised clinical trials to evaluate what is happening in routine clinical practice. These data are increasingly used to support regulatory decision-making and to guide clinical practice in real-world populations [1]. While the focus of evidence generation using RWD has traditionally been on clinical endpoints (safety and effectiveness outcomes), in order to provide a more holistic view of disease and well-being there is a need for RWD that capture the patient perspective.

A patient-centric paradigm shift has already occurred in the clinical trial domain, where patient-reported outcomes (PROs) are routinely integrated into trial design [2]. These data provide assessments of how a patient feels and functions at a given point; they are measured using standardised direct-to-patient questionnaires. Particularly in fields such as oncology, these data can be pivotal in helping to differentiate interventions in which clinical outcomes (such as survival) may appear comparable, and to provide additional data on the impact of a treatment beyond that which can be obtained from traditional endpoints (e.g. an assessment of tolerability). Furthermore, it is well-documented that a patient’s and clinician’s view of disease and well-being can differ substantially [3, 4], so these data provide valuable insight into patient experiences that might not otherwise be reported to or recorded by treating physicians, but that may have a meaningful impact on clinical outcomes [5–7].

Outside clinical trials, PRO measures can be incorporated into sources of RWD that are designed for research purposes, such as patient registries. The integration of PROs into prospective data capture is, however, resource intensive and maintaining patient engagement can be challenging, particularly in groups of patients who are older, sicker and of lower socioeconomic status [8]. Distinct from structured PROs, unstructured patient-generated health data (PGHD) are those data captured or recorded spontaneously by patients or their carers [9]. These data can be collected from a variety of sources including patient-powered research networks and smart wearable devices, as well as social media. Leveraging PGHD to generate insight into patient-experienced outcomes in the real world offers an exciting area for research, and is gaining attention from scientists, industry and regulators. Indeed, the US Food and Drug Administration (FDA) has recently encouraged the exploration of social media for this purpose [10]. The goal of this paper is to discuss the potential utility of social media as a unique source of PGHD to capture the patient perspective and patient-experienced outcomes in the real world.

## Harnessing social media for real-world data

Social media platforms such as Facebook, Twitter and patient networks have created abundant opportunities for patients and their carers to create and exchange health-related information. Previous work has found that patients tend to use social media platforms to increase knowledge, for social support, to exchange advice and to improve self-care and doctor–patient communication [11–13]. This has in turn generated a potentially rich but analytically ‘messy’ source of RWD; the ability to harness these data for medical research has been assisted in recent years by the application of advanced analytics. Approaches such as natural language processing coupled with machine learning are now able to effectively deal with the many complexities of the data extracted from social media, including multiplicity of terms, duplicate posts, misspellings and abbreviations (among others) [14]. Furthermore, in place of manual coding, machine learning algorithms can be developed which accurately and automatically identify features of posted content, such as adverse events (AEs), enabling the analysis of hundreds of thousands of text-based posts [15, 16]. Data can also be easily extracted from publicly available sites, bypassing many logistical hurdles associated with traditional approaches and allowing for accelerated, real-time and cost-effective data collection.

Pharmacovigilance in particular has been an area of early development in the utilisation of social media data. This is because, outside of clinical trials, more than 95% of treatment-related AEs are estimated to remain undocumented by healthcare professionals [17]. Because social media is adopted by patients to seek advice and share experiences, it is thought these data may enable greater capture of AEs, augment real-time reporting and in turn enable expedited signal detection. Indeed, approximately 12–62% of all posts on patient forums have been found to include information related to an AE [18]. Initial work has explored the extent to which these data correspond with existing pharmacovigilance sources, and a recent systematic review found good concordance (between 57% and 99%) for AEs reported in social media [19]. Although concordance is generally good [20], where differences have been observed it has been found that social media data tend to include a higher frequency of AEs relating to milder, unpleasant or quality of life events, with severe events requiring clinical diagnosis being underrepresented [17]. However, it is important to consider that rather than being a limitation with respect to the validity of the data, these differences may instead reflect nuances in data capture. Indeed, other work has shown that patient and clinical agreement tends to be higher for observable symptoms but poorer for subjectively experienced symptoms such as fatigue [21]. By integrating the patient perspective, PGHD from social media may offer additional dimensionality to the routine monitoring of drug safety, as well as more broadly capture symptoms or experiences relevant to patients that may otherwise remain under-recorded. Reflecting the potential importance of social media data for pharmacovigilance, the US FDA signed an agreement in 2015 with PatientsLikeMe (a patient network) to determine how patient-reported data from the platform could help to generate insight into drug safety [22].

Beyond pharmacovigilance, other studies have shown that social media data can be used meaningfully to understand patient experience with their disease or treatment more broadly. For example, a recent study extracted >10,000 data points from a variety of social media platforms and developed a machine learning algorithm to automatically identify mentions of treatment switching among patients with multiple sclerosis. The most common reasons for switching were then mapped and found to be comparable to those obtained from published data [23]. Sentiment analysis is another promising area for this type of social media analytics [24]. This approach involves assessing the ratio of positive to negative words contained in a post to ascribe positive, negative or neutral sentiment to opinion-based text. This approach has previously been applied to understand experience with systemic treatment options among patients with multiple sclerosis [25], attitudes towards vaccinations [26, 27], and to monitor mood among cancer patients online [28]. More traditional qualitative content analysis can also be applied to extracted text from social media, albeit on a smaller scale owing to the manual nature of these techniques. This approach has also been successfully applied, for example, to understand patient perception of care quality [29].

## Potential limitations and challenges

Despite a number of potential applications, using social media to capture the patient perspective is not without challenges. Exploration of topics can often be limited; Twitter, for example, only allows individuals to write 280 characters. Many discussions also take place in private patient forums, largely inaccessible to researchers. Although analytical techniques to deal with complexities inherent in social media data continue to advance, it may often be the case that there is too much noise to generate meaningful insight.

Beyond technical issues surrounding data capture, issues concerning the representativeness of the patient population are also important. Indeed, the demographics of individuals posting on social media are rarely known. Where it is possible to garner this information, data show active users tend to be younger, women, more highly educated and less acutely ill or functionally impaired [30, 31], presenting issues of external validity. Indeed, the ‘digital divide’ in Internet usage has been well documented; although recent reports suggest Internet usage in adults aged over 65 years has doubled in recent decades, older adults (>75 years) and those with functional impairment remain less likely to engage in health-related Internet usage [31]. It is also possible that, for older adults, younger carers or relatives may be engaging online on the patient’s behalf. It is essential to quantify demographic disparities in order to apply analytical strategies that help mitigate biases in patient representativeness (e.g. stratified sampling). Identifying proxies for demographic information is one potential solution for this; recent work has used machine learning techniques to show that features extracted from patients’ user names can be used to accurately infer patient demographics [32].

There may also be nuances in the data captured within social media. For studies that have attempted to validate data from social media with data obtained from traditional sources, further investigation is needed into the extent to which observed differences reflect issues in data quality (e.g. as a result of the limited representation of certain groups) as opposed to more general intricacies in the type of information patients may be more likely to share in online communities (e.g. quality of life events). Importantly, the current notion is not that social media should replace existing patient-reported data, but rather that the observed benefits of these data (rapid, cost-effective and large-scale access to real-world PGHD) should be harnessed to complement existing data sources. However, as the world continues to get more and more connected this needs to be continually assessed.

Privacy concerns also remain a fundamental challenge and a barrier to effectively harnessing social media data for public health. Even though text extraction takes place on content that is posted ‘publicly’, it can be contested whether or not it is correct to presume consent for the use of these data. Other publications have provided more detailed discussions regarding the ethical considerations associated with using these data [33, 34]. It should nonetheless be noted that privacy concerns are not unique to social media and are seen in other areas in which patient data are used for public health research or surveillance [35]. In these domains, effective communication and patient engagement are known to be key [36]. Indeed, studies have shown that the more patients know about how their data are used, the more accepting they are of data sharing [37, 38]. The same considerations will likely apply when targeting patient acceptability for social media. Encouragingly, early data show good acceptability, with 71% of patients recruited at emergency departments in the US identified as being willing to share their social media data for public health research [30].

## Future perspective

As the science for extracting and analysing data from social media continues to advance, there are a number of interesting future applications which may further extend the utility of these data. For example, some initial work using machine learning algorithms has shown that it is possible to predict a diagnosis of depression recorded in a patient’s medical record up to 6 months prior using only the language content of their Facebook posts [39]. Other studies have shown similar feasibility for detecting depression using only data from Twitter [40]. The implication is that these data could be used in the future to facilitate a scalable screening tool for detecting mental illness. Of course, there are ethical and regulatory logistical challenges that would need to be addressed in order to effectively implement such a programme. However, that data posted on social media could identify patients who may benefit from targeted interventions who remain undetected because they fail to present to or discuss symptoms with their clinician is an exciting area for development. Equally, as patients continue to use social media to seek information related to their health, data from social media could be used to develop patient-focused strategies or interventions aimed at better supporting patients’ needs by providing targeted information and support.

Linking social media profiles to electronic medical records may also offer an opportunity to further extend the utility of these data in the future [13]. From an epidemiological perspective, this would allow background demographic and health information to be captured for these digital cohorts. In turn, it would allow analyses to be extended to include, for example, comparative effectiveness research. From a care perspective, these data could assist with improved patient-centred management. For example, AEs reported on social media could be communicated back to healthcare professionals. In doing do, these data could provide a means by which to encourage more open and sustained patient–clinician communication, a concept fundamental to patient-centred care [41]. There are of course challenges associated with data extraction and linkage that would need to be overcome before this next-generation of health-enabled social media can be realised, but this reflects an exciting area of future research.

## Conclusion

As patients increasingly turn to social media as a means of seeking information or sharing experiences, these data offer a unique opportunity to capture patient-generated data in the real world. The feasibility of harnessing social media data has been assisted in recent years by the evolution of advanced analytics. The ability to generate insights from these data relevant to public health has already been demonstrated with some success. Although there are a number of exciting potential future applications of these data, privacy and governance considerations remain a fundamental concern for advancement of the field.

## Abbreviations

AE: Adverse events
FDA: Food and Drug Administration
PGHD: Patient-generated health data
PRO: Patient-reported outcome
RWD: Real-world data
